# Renal pelvis metastasis following surgery for breast angiosarcoma: a case report and literature review

**DOI:** 10.3389/fonc.2024.1296328

**Published:** 2024-03-21

**Authors:** Fuyu Guo, Shiwei Sun, Xiangnan Niu, Yue Wang, Wei Yao, Peng Yue, Xiaoqian Deng, Jiwen Shang, Yangang Zhang

**Affiliations:** ^1^ Third Hospital of Shanxi Medical University, Shanxi Bethune Hospital, Shanxi Academy of Medical Sciences, Tongji Shanxi Hospital, Taiyuan, China; ^2^ Department of Urology, Taiyuan Central Hospital of Shanxi Medical University, Taiyuan, China; ^3^ Shanxi Bethune Hospital, Shanxi Academy of Medical Sciences, Tongji Shanxi Hospital, Third Hospital of Shanxi Medical University, Taiyuan, China; ^4^ Tongji Hospital, Tongji Medical College, Huazhong University of Science and Technology, Wuhan, China

**Keywords:** breast angiosarcoma, renal pelvis cancer, tumor metastasis, diagnosis, treatment, robot-assisted laparoscopic surgery

## Abstract

Renal metastasis of breast angiosarcoma is rare. This article reports the medical records of a patient diagnosed with breast angiosarcoma who underwent radical mastectomy and was found to have multiple lung metastases 3 years after surgery and renal pelvic metastasis 4 years after surgery. The patient underwent robot-assisted laparoscopic radical nephroureterectomy and sleeve resection of the intramural segment of the ureter, and postoperative pathology and immunohistochemical staining confirmed the diagnosis of renal pelvic metastasis of breast angiosarcoma. The patient received anlotinib for lung metastases following surgery and was followed up for 4 months after surgery. Currently, the patient has symptoms of coughing and hemoptysis but no other discomfort. The diagnosis and treatment of this rare malignant tumor remain challenging.

## 1 Introduction

Breast angiosarcoma (BA) is a highly invasive malignant tumor that originates from the endothelial cells of the breast blood vessels. It is most common in women aged 20-40 years and accounts for less than 1% of all breast cancers. The 5-year overall survival rate is only around 30% (1, 2). BA can metastasize hematogenously to the contralateral breast, lungs, bones, liver, ovaries, skin, and subcutaneous tissues, whereas metastasis to the orbital tissues and lymph nodes is rare (3, 4). Primary breast angiosarcoma (PBA) often presents as a rapidly enlarged round-like mass located in the depths of the breast in a short period of time, which can show traumatic purplish-red changes when the tumor is superficial or invades the skin (1, 5). However, there is currently no literature on PBA that has metastasized to the kidney or renal pelvis. Here, we describe the diagnosis and treatment of PBA in a 33-year-old female.

## 2 Case description

**Figure 1:**
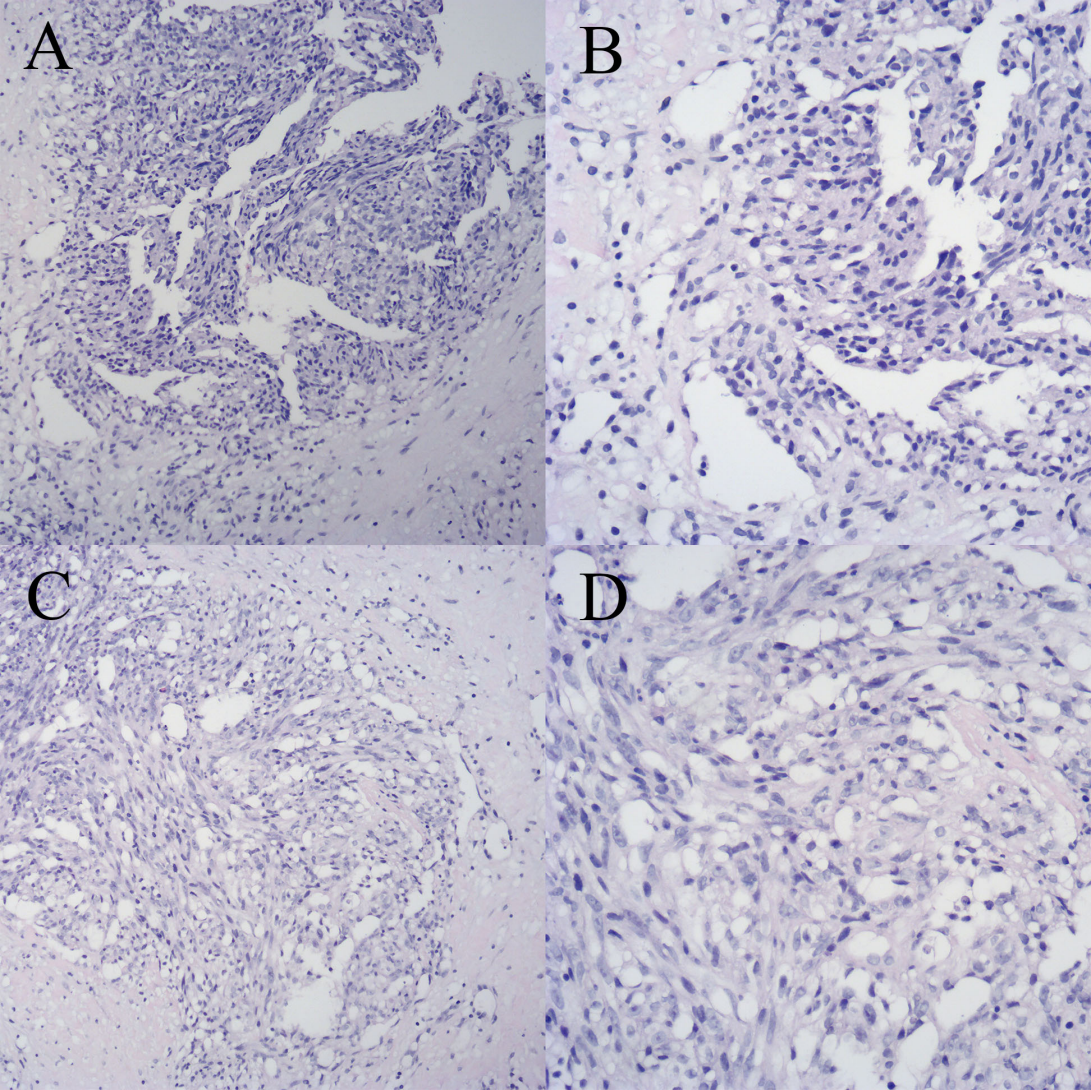
Breast biopsy specimen with hematoxylin and eosin (HE) staining. **(A)** Specimen 1 (×100). **(B)** Specimen 1 (×200). **(C)** Specimen 2 (×100). **(D)** Specimen 2 (×200).

**Figure 2:**
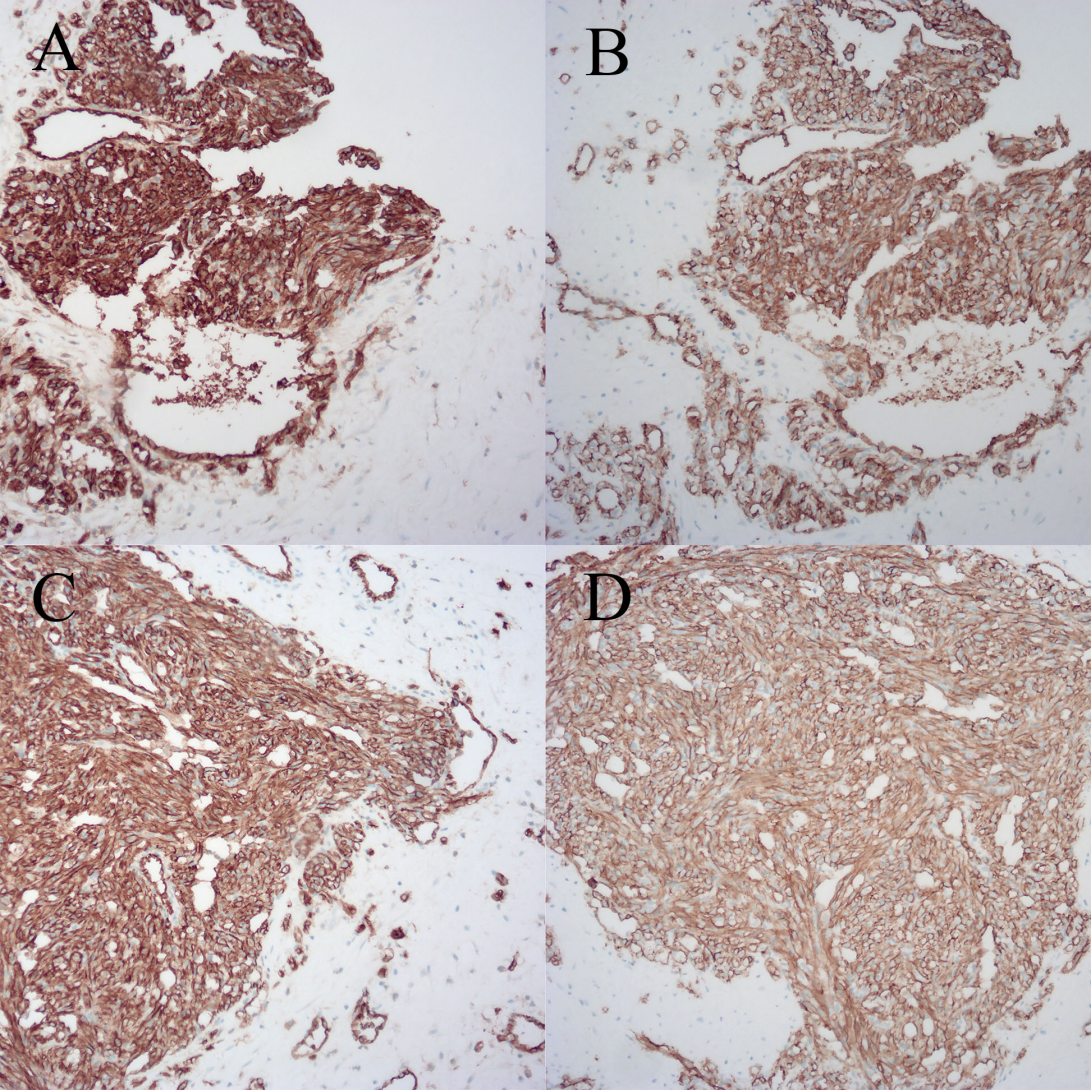
Breast biopsy specimen with immunohistochemical staining. **(A)** Specimen 1, CD31. **(B)** Specimen 1, CD34. **(C)** Specimen 2, CD31. **(D)** Specimen 2, CD34.

The patient is a 33-year-old female, with no family history of breast tumor. In October 2019, she underwent needle biopsy of a right breast mass and was diagnosed with PBA (**Figure 1**). Immunohistochemical staining showed AE1/AE3 (- [Indicates a negative result]), CK7 (-), GATA3 (-), Vimentin (+ [Indicates a positive result]), CD34 (+), CD31 (+), SMA (-), calponin (-), ER (-), PR (-), CerbB-2 (0), β-catenin (cytoplasmic +), CD10 (-), and Ki67 (approximately 70% +) (**Figure 2**). The patient received radical mastectomy and paclitaxel for 10 cycles. In January 2022, due to coughing and bloody sputum, she underwent computed tomography (CT) of the lungs and needle biopsy; she was subsequently diagnosed with multiple lung metastases. The patient received targeted therapy and showed a poor response to apatinib but achieved good control after switching to anlotinib.

## 3 Diagnostic assessment

On November 15, 2022, the patient was admitted to the hospital due to gross hematuria for 10 h, accompanied by dizziness and fatigue. The blood cell analysis revealed hemoglobin (Hb) at 76 g/L and a red blood cell (RBC) count of 2.45 × 10¹²/L. Urinalysis showed an entire field of RBCs. The chest CT scan showed multiple solid nodules in both lungs, with the larger one located in the apicoposterior segment of the upper lobe of the left lung, measuring approximately 1.79 × 1.39 cm. The edge of the nodule was lobulated and uniformly enhanced on a contrast-enhanced scan. Abdominal CT suggested destruction of the structure of the right renal pelvis and calyces, with a lump-like and slightly high-density shadow in the renal sinus area, with the largest cross-sectional size approximately 4.46 × 4.81 cm. Contrast-enhanced scanning revealed multiple irregularly enhanced areas inside the mass in the arterial phase with multiple small blood vessels visible. The adjacent renal parenchyma exhibited decreased enhancement. CT revealed a malignant tumor in the right renal pelvis that had metastasized from other origins, or a bleeding pseudoaneurysm (**Supplementary Figures 1, 2**). Cystoscopy revealed no ureteral bleeding, and a biopsy of the bladder tissue revealed chronic inflammation of the mucosa. Urine cytology results were grade II and urine fluorescence *in situ* hybridization (FISH) was negative. Owing to the patient’s wishes, no further treatment was administered.

**Figure 3:**
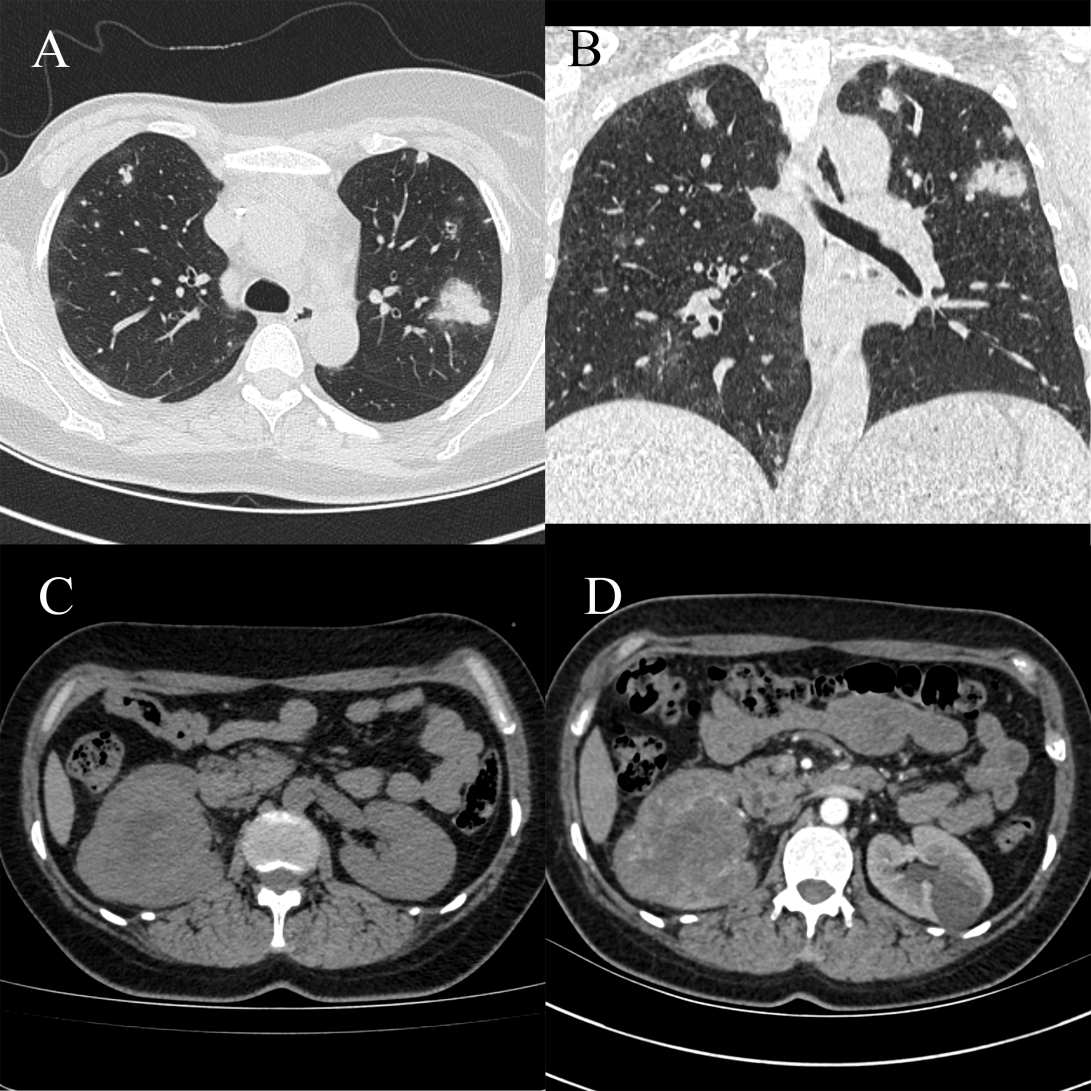
Computed tomography (CT) scan showing an increase in the number and size of lung lesions compared to previous results, with a slight increase in the right renal sinus mass. **(A)** Chest CT scan, horizontal position. **(B)** Chest CT scan, coronal position. **(C)** Abdominal CT scan, horizontal position. **(D)** Abdominal CT scan, arterial phase, horizontal position.

After 3 months, CT showed an increase in the number and size of the lung lesions, and the tumor in the right renal pelvis was slightly larger, with the largest cross-sectional size of approximately 6.60 × 8.10 cm (**Figure 3**). Renal angiographic ultrasound showed that the tumor began to enhance at 17 s and then rapidly increased as a whole, with no enhancement visible inside the tumor throughout, reaching a peak at 20 s with high enhancement, and subsiding slightly earlier than the surrounding renal parenchyma (**Supplementary Figure 3**). The patient underwent robot-assisted laparoscopic right nephroureterectomy and transurethral cystoscopy for bladder clot removal (**Supplementary Figure 4**). Bleeding was observed at the right ureteral orifice during surgery. Postoperative pathology showed a tumor measuring 7.00 × 5.50 × 3.70 cm in the right renal pelvis, with cancerous tissue penetrating the perirenal fat and visible cancerous tissue in the blood vessels. Immunohistochemical staining showed CK20 (-), 34βE12 (-), β-catenin (membrane and cytoplasmic +), p63 (-), GATA-3 (focally weak +), S100P (-), Uroplakin II (-), CK7 (-), Ki67 (approximately 70% +), AE1/AE3 (-), EMA (-), Vimentin (+), CD31 (partial +), CD34 (+), SMA (-), Desmin (-), PAX-8 (-), INI-1 (expression), OCT3/4 (-), ALK D5F3 (-), CyclinD1 (partial +), HMB45 (-), Melan-A (partial +), S100 (-), Myogenin (-), Fli-1 (+), TLEl (+), and CD99 (partial cytoplasmic +) (**Supplementary Figures 5, 6**). The pathological diagnosis was consistent with angiosarcoma, partially presenting with an epithelial-like morphology. Follow-up at 4 months postoperatively showed symptoms of coughing and hemoptysis, with no other discomfort. CT revealed no abnormalities in the surgical area of the right kidney, but showed aggravation of lung metastasis (**Supplementary Figure 7**).

## 4 Discussion

BA is a rare malignant tumor. There are only a few retrospective studies and case reports but no large randomized controlled trials. Therefore, there are still no clear guidelines and evidence to guide the treatment of this disease. Surgery is still the main treatment at present (1). Due to the aggressive and multifocal nature of angiosarcoma, the prognosis is poor when the margins are positive (6). Currently, breast angiosarcoma is mainly treated with mastectomy to achieve as negative a margin as possible, and the recurrence rate after breast-conserving surgery is high (7, 8). There is still no definite evidence that adjuvant chemotherapy and radiotherapy can improve patient survival.

Angiosarcoma may present local recurrence and distant metastasis in the early stage, and the prognosis is significantly worse than that of other breast tumors. Studies have shown that DFS and OS of low - and medium-grade breast tumors are significantly longer than those of high-grade tumors (7, 9). PBA is prone to metastasize to the contralateral breast, lungs, bones, liver, ovaries, skin, and subcutaneous tissues hematogenously. However, there is currently no record of metastasis to the kidneys or renal pelvis. This disease can be differentiated from the following conditions.

Primary carcinoma of the renal pelvis: This is a subtype of urothelial carcinoma. Ureteroscopy and histopathological examinations are the gold standard for this form of carcinoma. Urine cytology and urine FISH examination are economical and convenient non-invasive methods with good sensitivity and specificity (10). Immunohistochemical (IHC) Staining is an application of immunostaining. It is based on antigen-antibody reaction, where it uses antibodies to determine the tissue distribution of an antigen of interest, such as tumor antigens. IHC is an important tool in diagnostic and research. The most important markers for malignant urothelial carcinoma are GATA3, CK7, CK20, and p63 (11), whereas for angiosarcoma, CD31 is the most specific marker for endothelial cell differentiation, and CD34 is more sensitive. Ki-67 reflects the mitotic rate of cells and is higher in sarcomas with invasive characteristics (12). The transcription factors ETS-related gene (ERG) and friend leukemia integration 1 (FLI-1) are new antibodies with high sensitivity and specificity compared with CD34 and CD31 (13).

Renal pelvic hematoma, otherwise known as renal pseudoaneurysm, is a renal pseudotumor caused by the rupture and bleeding of small renal parenchymal arteries. CT scans showed a slightly high-density mass with a clear boundary from the renal parenchyma, which could cause compression of the renal pelvis and calyces without signs of destruction. In the acute or subacute phase, the density of the mass may increase slightly owing to the entry of contrast agents into the lesion through the ruptured blood vessels.

Renal artery aneurysm: On CT, it appears as a uniform, slightly high-density mass with clear edges and calcifications on the wall. The tumor body was clearly enhanced. The difference is that the degree of aneurysm enhancement is the same as that of the abdominal aorta and renal artery at any time, and renal artery angiography remains the gold standard for diagnosis and treatment (14).

Nephroureterectomy is the gold standard treatment for upper urinary tract tumors, including total resection of the affected kidney and ureter to the ureteral orifice. Due to the long learning time for laparoscopic techniques, high surgical difficulty, and high tension in bladder incision suture reconstruction, its further promotion and application are limited. Robot-assisted laparoscopy has many advantages over traditional laparoscopy, including a three-dimensional high-definition surgical view, simulated arms with seven degrees of freedom, and the ability to automatically filter hand tremors (15). Currently, there are few reports of single-position robot-assisted laparoscopic nephroureterectomy for renal pelvic carcinoma in China. In this case, the patient had a minor intraoperative injury, good postoperative recovery, and no adverse complications.

This study is the first reported case of renal metastasis from a PBA to date. The diagnosis and treatment of this rare malignant tumor remain challenging.

## 5 Patient perspective

The patient is satisfied with the treatment effect. The personal information related to the patient has been hidden from this manuscript. Written informed consent was obtained from the individual(s), and minor(s)’ legal guardian/next of kin, for the publication of any potentially identifiable images or data included in this article.

## Data availability statement

The original contributions presented in the study are included in the article/**Supplementary Material**. Further inquiries can be directed to the corresponding author.

## Ethics statement

Written informed consent was obtained from the individual(s), and minor(s)’ legal guardian/next of kin, for the publication of any potentially identifiable images or data included in this article.

## Author contributions

FG: Conceptualization, Data curation, Project administration, Writing – original draft. SS: Conceptualization, Data curation, Writing – original draft. XN: Conceptualization, Data curation, Writing – original draft. YW: Data curation, Writing – original draft. WY: Data curation, Writing – original draft. PY: Data curation, Writing – original draft. XD: Data curation, Writing – original draft. JS: Supervision, Writing – review & editing. YZ: Conceptualization, Project administration, Supervision, Writing – review & editing.
