# Supplementary material for: Renal pelvis metastasis following surgery for breast angiosarcoma: a case report and literature review
Source: Front Oncol. 2024 Mar 21;14:1296328. doi: 10.3389/fonc.2024.1296328 (PMC10991732; doi:10.3389/fonc.2024.1296328)
Supplement: Supplementary file 1 [file Image_1.pdf]

Supplementary Figure Legends

Supplementary Figure S1. Chest computed tomography (CT) scan showing multiple solid nodules in both lungs, with the larger one located in the posterior segment of the left upper lobe.

a, b. Horizontal position

c. Coronal position

d. Sagittal position

Supplementary Figure S2. Abdominal computed tomography (CT) scan showing a slightly high-density mass in the right renal sinus area with multiple irregular enhanced areas inside.

a. Plain scan, horizontal position

b. Arterial phase, horizontal position

c. Plain scan, coronal position

d. Arterial phase, coronal position

Supplementary Figure S3. Renal angiography ultrasound.

Supplementary Figure S4. Robot-assisted laparoscopic right nephroureterectomy

a. Clamping of renal blood vessels

b. Dissection of the kidney

c. Dissection of the ureter

d. Search for the ureter-bladder anastomosis site

Supplementary Figure S5. Renal pelvis surgical specimen staining

a. HE ×40

b. HE ×100

c. Immunohistochemical staining GATA-3

d. Immunohistochemical staining Uroplakin II

Supplementary Figure S6. Renal pelvis surgical specimen immunohistochemical staining

a. CD31

b. CD34

c. AE1/AE3

Supplementary Figure S7. Computed tomography (CT) scan showing worsening of lung metastasis compared to previous results, with changes in the right kidney after surgery and a locally visible encapsulated fluid area.

a. Chest CT scan, horizontal position

b. Chest CT scan, coronal position

c. Abdominal CT scan, horizontal position

d. Abdominal CT scan, coronal position

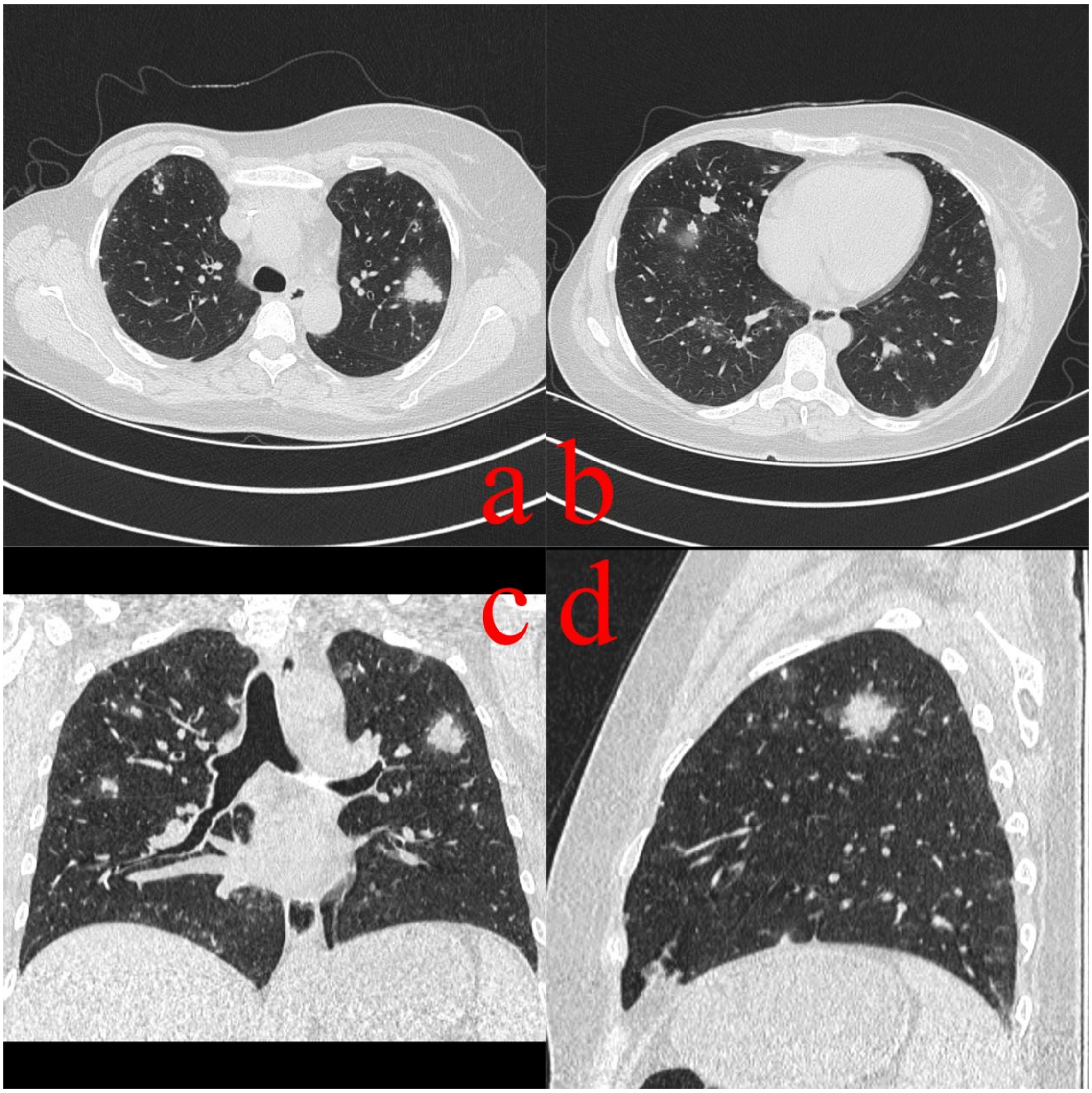

Supplementary Figure S1. Chest computed tomography (CT) scan showing multiple solid nodules in both lungs, with the larger one located in the posterior segment of the left upper lobe.

a, b. Horizontal position

c. Coronal position

d. Sagittal position

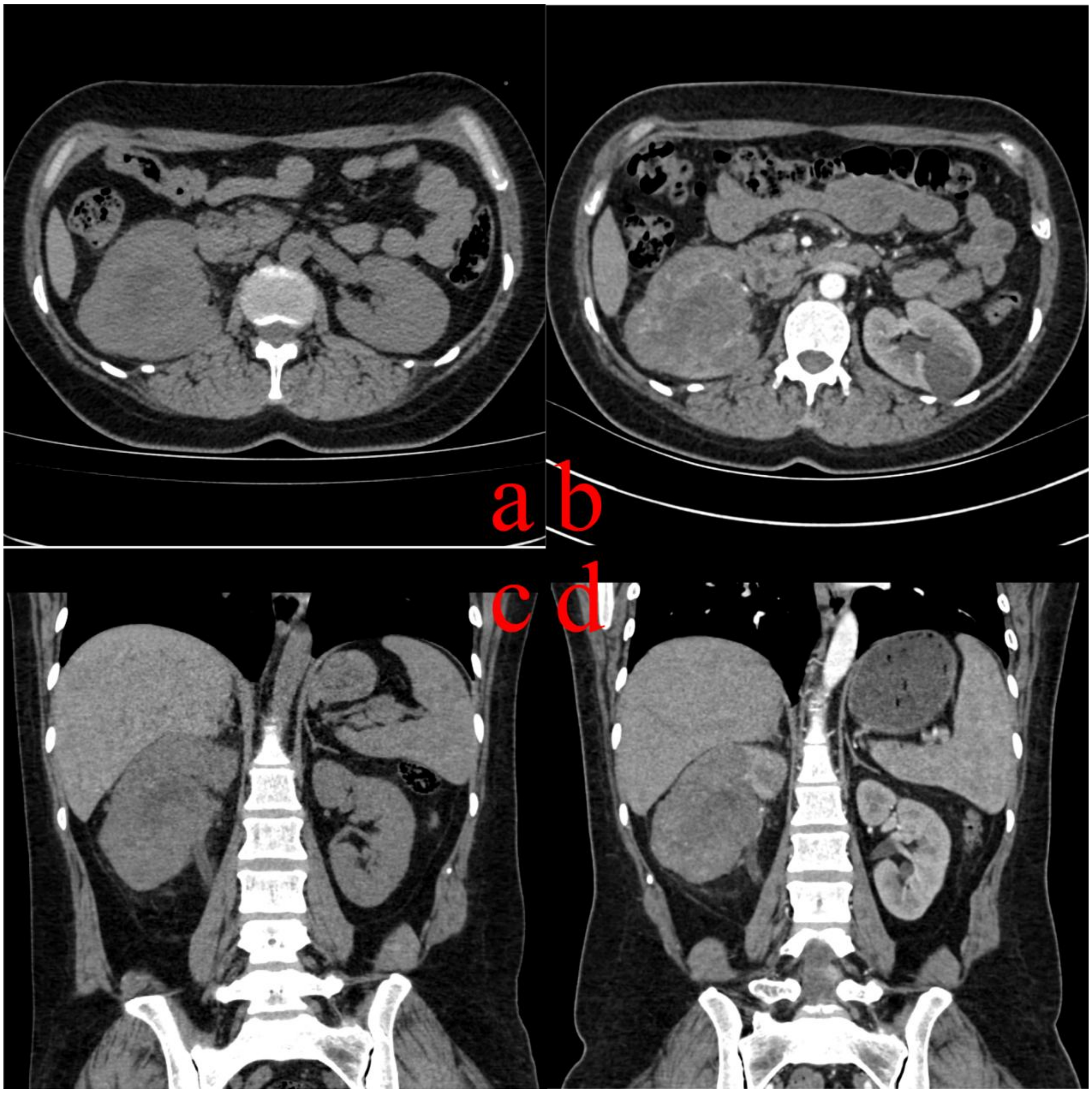

Supplementary Figure S2. Abdominal computed tomography (CT) scan showing a slightly high-density mass in the right renal sinus area with multiple irregular enhanced areas inside.

a. Plain scan, horizontal position

b. Arterial phase, horizontal position

c. Plain scan, coronal position

d. Arterial phase, coronal position

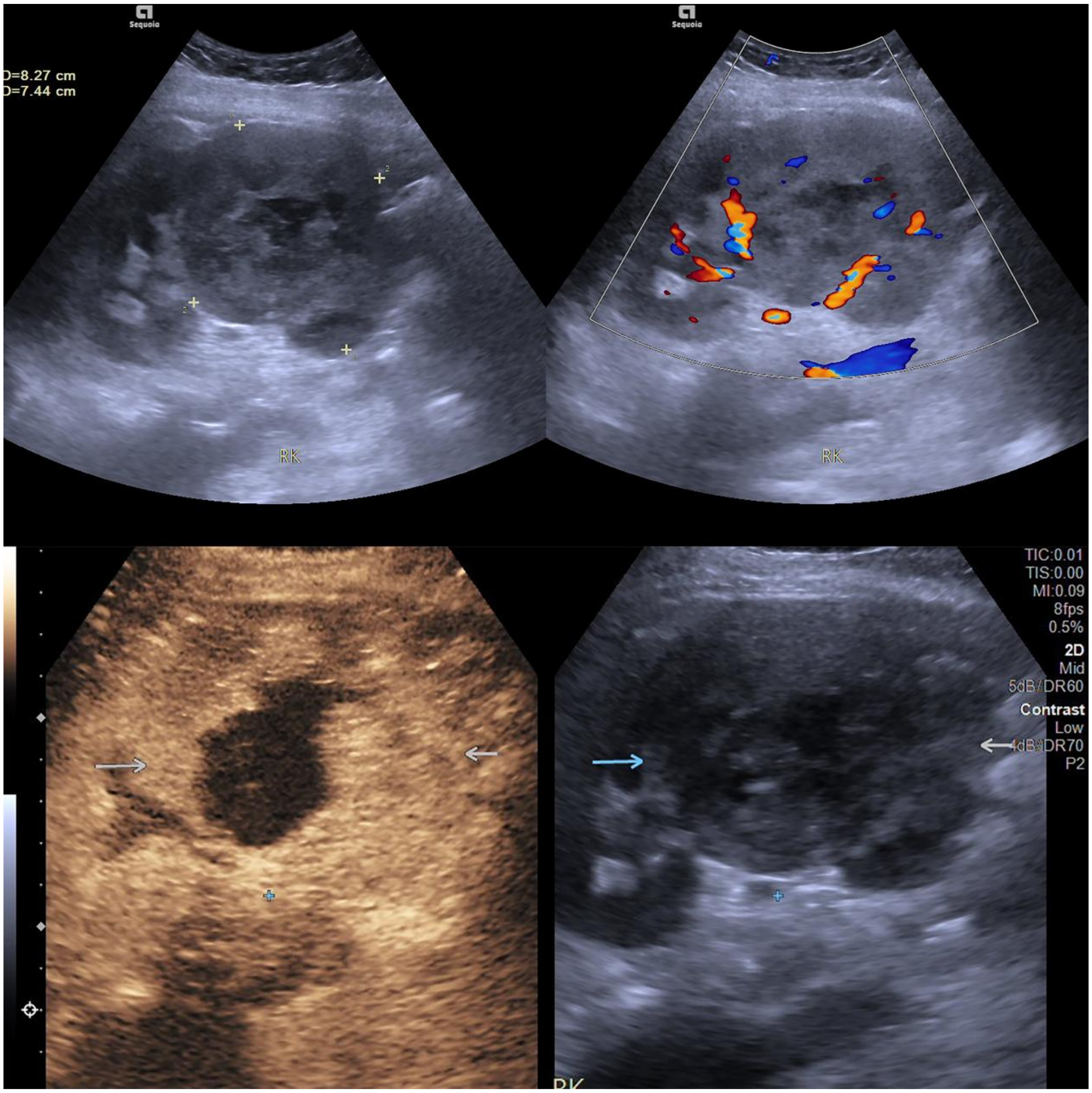

Supplementary Figure S3. Renal angiography ultrasound.

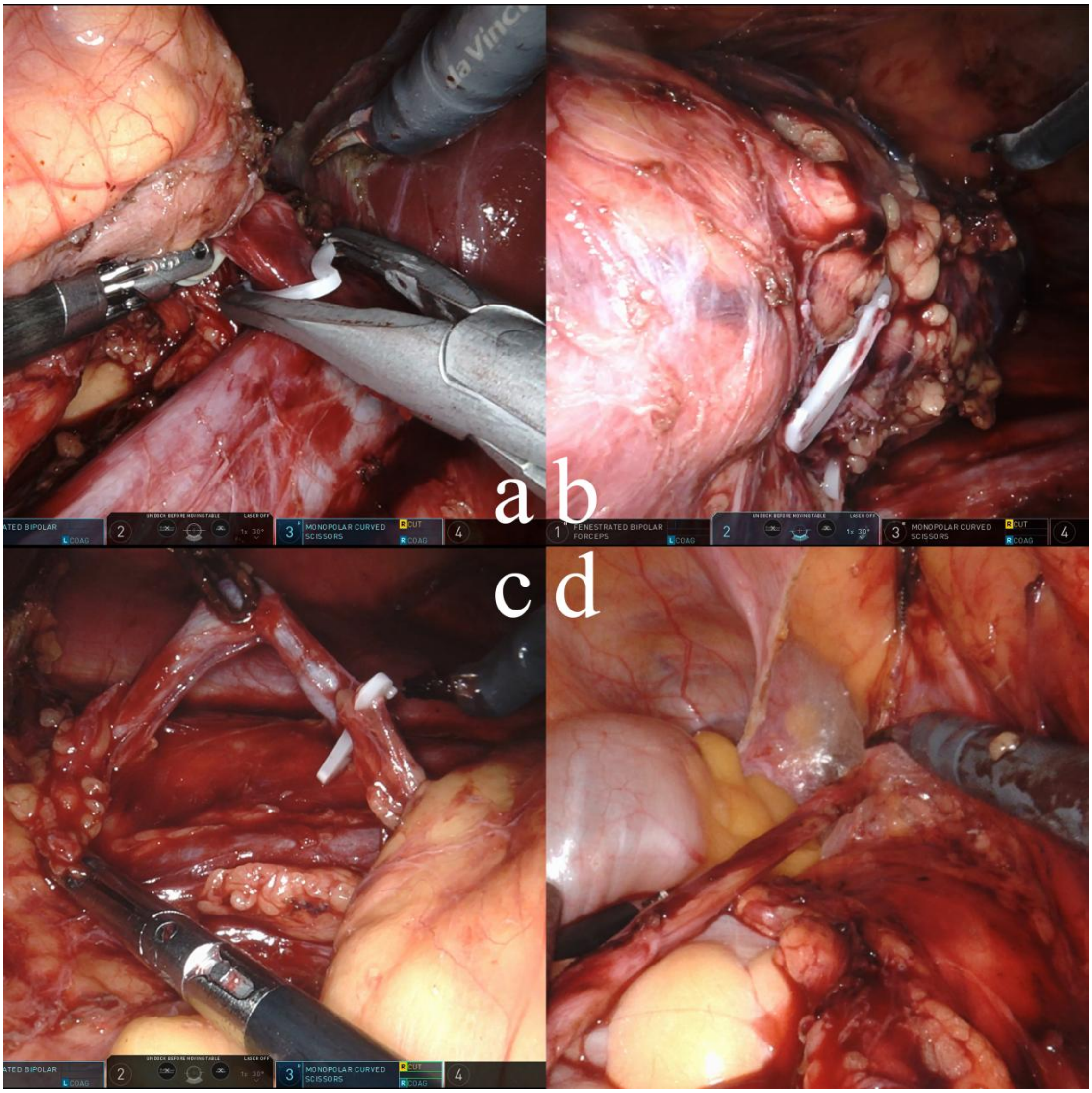

Supplementary Figure S4. Robot-assisted laparoscopic right nephroureterectomy

a. Clamping of renal blood vessels

b. Dissection of the kidney

c. Dissection of the ureter

d. Search for the ureter-bladder anastomosis site

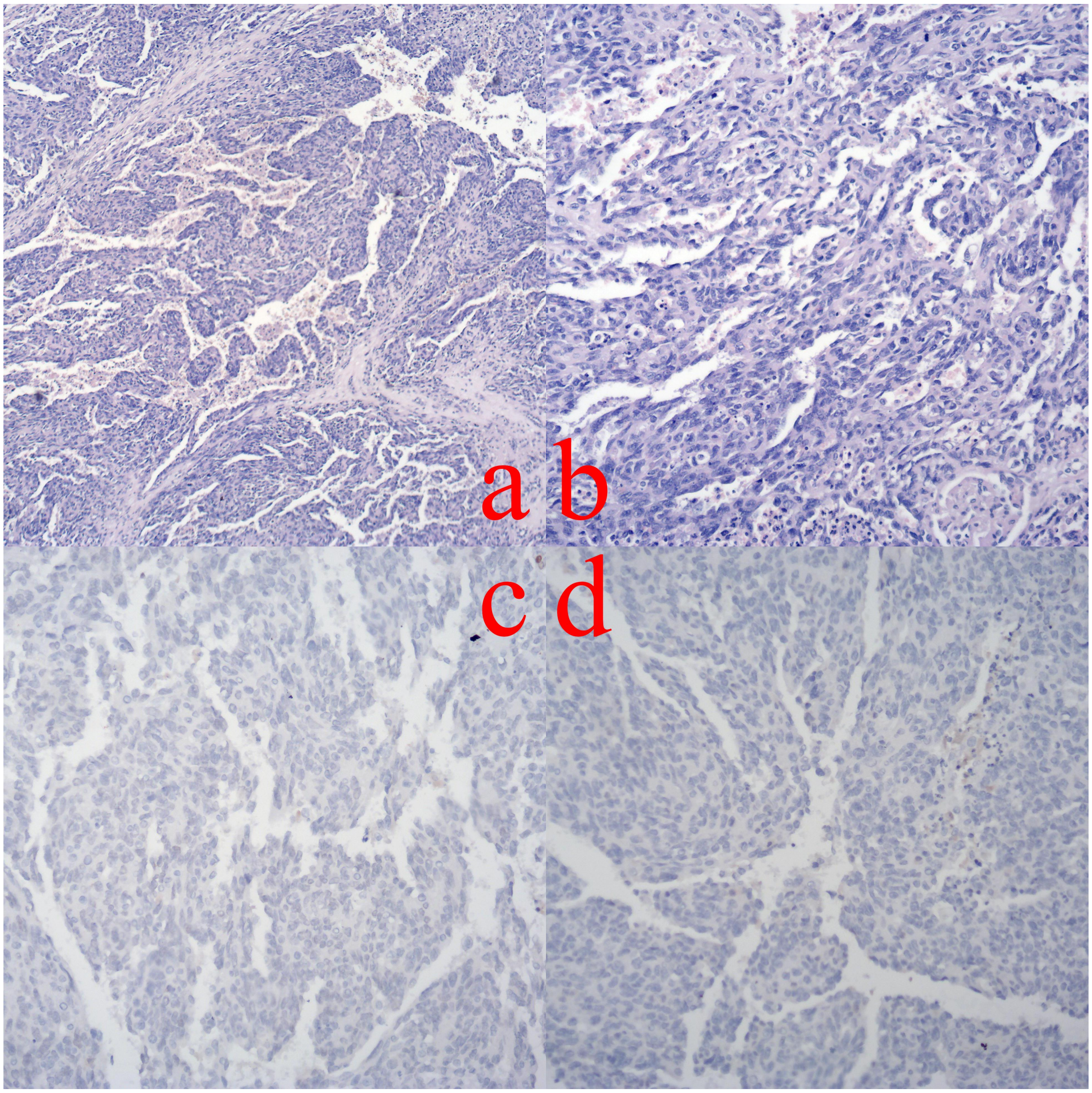

Supplementary Figure S5. Renal pelvis surgical specimen staining

a. HE ×40

b. HE ×100

c. Immunohistochemical staining GATA-3

d. Immunohistochemical staining Uroplakin II

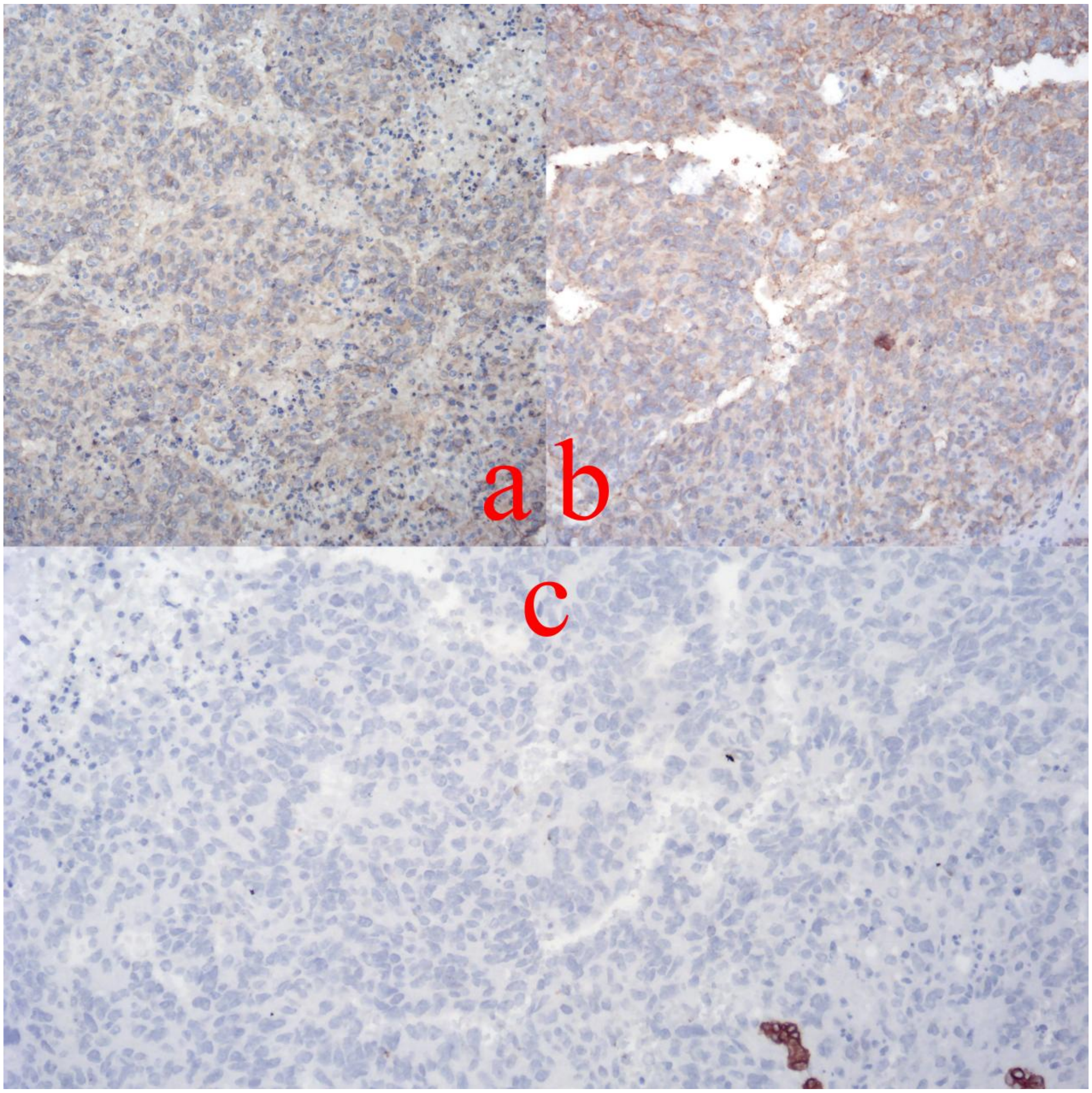

Supplementary Figure S6. Renal pelvis surgical specimen immunohistochemical staining

a. CD31

b. CD34

c. AE1/AE3

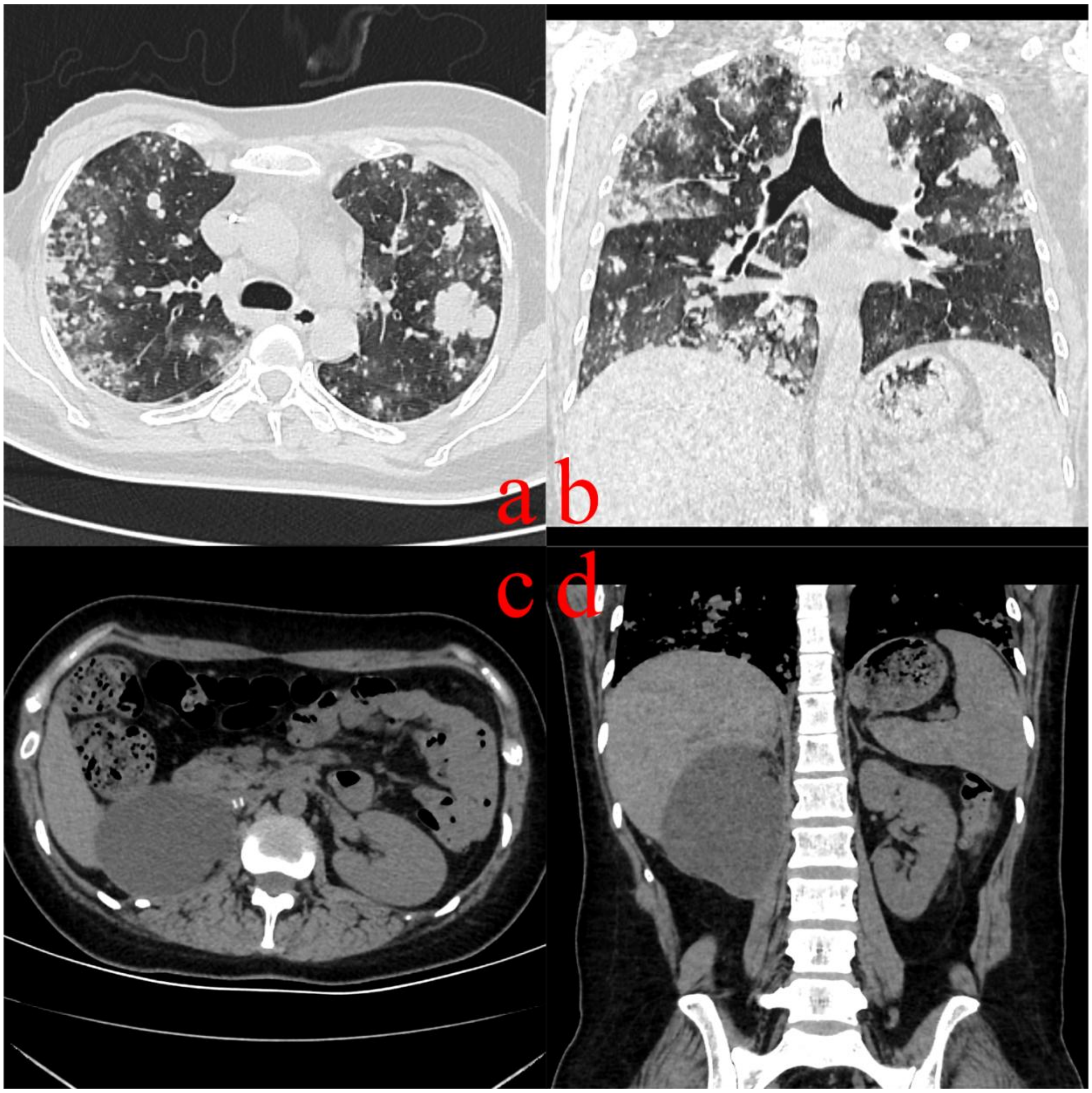

Supplementary Figure S7. Computed tomography (CT) scan showing worsening of lung metastasis compared to previous results, with changes in the right kidney after surgery and a locally visible encapsulated fluid area.

a. Chest CT scan, horizontal position

b. Chest CT scan, coronal position

c. Abdominal CT scan, horizontal position

d. Abdominal CT scan, coronal position
